# Canada’s First Joint Oncology-Allergy Clinic: Successful Desensitization to Trastuzumab Following Severe Anaphylactic Reaction in Which Epinephrine Was Inappropriately Withheld

**DOI:** 10.3390/curroncol30030218

**Published:** 2023-02-27

**Authors:** Madeline Robinson, Marc Geirnaert, Brady Anderson, Lundy McKibbin

**Affiliations:** 1 Max Rady College of Medicine, University of Manitoba, Winnipeg, MB R3E 0W2, Canada; 2 Provincial Oncology Drug Program, CancerCare Manitoba, Winnipeg, MB R3E 0V9, Canada; 3 Section of Medical Oncology, Department of Internal Medicine, University of Manitoba, Winnipeg, MB R3E 0W2, Canada; 4 University of Manitoba, Department of Internal Medicine, Section of Allergy & Clinical Immunology, Winnipeg, MB R3E 0W2, Canada

**Keywords:** oncology, allergy, immunology, anaphylaxis, hypersensitivity, desensitization, trastuzumab, chemotherapy, monoclonal-antibody, multidisciplinary

## Abstract

Background: Recognition of anaphylaxis and differentiation from other infusion reactions in an oncology setting is imperative; epinephrine is the recommended treatment for anaphylaxis and should be administered immediately to patients in whom anaphylaxis is suspected. Trastuzumab has a potentially tremendous oncological benefit, and when hypersensitivity reactions occur, rechallenge with desensitization protocols has become more common. Oncology presents a unique situation in which repeat drug exposure after a serious adverse reaction is often warranted due to the mortality risk of untreated cancer—allergists can assist with both symptom assessment and risk mitigation. Case presentation: This case showcases successful desensitization in a 43-year-old female with locally advanced HER2-positive breast cancer following a severe anaphylactic reaction to trastuzumab, in which epinephrine was not administered. We report the establishment of the Medical Oncology and Allergy Clinic: Canada’s first multidisciplinary clinic aimed at expediting the assessment and management of oncology patients with adverse drug reactions (including chemotherapy, contrast media, antimicrobials) and those with primary and acquired immunodeficiency. Conclusions: We propose this multidisciplinary clinic model as a treatment framework moving forward, with the goal of continuing first-line therapies in cancer patients who develop drug-hypersensitivity (i.e., through desensitization). This case highlights the unmet need for a multidisciplinary approach to the management of oncology patients who experience hypersensitivity reactions.

## 1. Introduction

Trastuzumab is a humanized recombinant monoclonal antibody agent targeting the proto-oncogene human epidermal growth factor receptor 2 (HER2) [1]. HER2 is overexpressed in approximately 20% of breast cancers [1]. Neoadjuvant trastuzumab has been shown to increase pathologic complete response rates when combined with chemotherapy, can cause regression of disease, and improve overall survival in patients with metastatic HER2-positive breast cancer [1,2]. 

While up to 40% of patients may experience trastuzumab-associated infusion reactions, severe hypersensitivity reactions occur in only 1–3% of patients [3]. Various mechanisms can cause acute reactions to monoclonal antibody therapies, such as trastuzumab, including IgE-mediated anaphylactic and anaphylactoid reactions, serum sickness, cytokine release syndrome, and tumor lysis syndrome [4]. Symptoms can range from local skin reactions to potentially fatal anaphylaxis [4]. Anaphylaxis is an acute multi-system allergic reaction—it is clinically diagnosed using one of three accepted diagnostic criteria [5]. Other tests can be used to support diagnosis in patients with atypical symptoms. Current guidelines recommend measuring serum tryptase from one-half to two hours after the onset of anaphylaxis, in addition to measurement of baseline tryptase 24 h after resolution of all anaphylaxis symptoms [5]. During anaphylaxis, serum tryptase concentrations increase as a result of mast cell degranulation, and while measuring levels during a clinical emergency will not help diagnose anaphylaxis, if elevated above baseline, it can be helpful in confirming the diagnosis of anaphylaxis retrospectively [5]. A serum tryptase 1.2 times higher than baseline tryptase +2 μg/L supports a diagnosis of anaphylaxis [5]. The baseline tryptase also permits the exclusion of other conditions associated with an elevated tryptase that can contribute to severe anaphylactic reactions, such as Mastocytosis and Hereditary Alpha Tryptasemia [5,6]. Skin-prick or intradermal testing post-reaction can also be conducted to evaluate for IgE-mediated disease when used in correlation with clinical history [7]. 

Epinephrine is the recommended treatment for anaphylaxis and should be administered immediately to patients in whom anaphylaxis is suspected [8]. This recommendation stands even if the diagnosis is uncertain, as there are no absolute contraindications to epinephrine administration in anaphylaxis [8]. Supportive therapy may also be indicated, including volume replacement with intravenous fluids, oxygen therapy, bronchodilators (inhaled β2-agonists), and antihistamines for the treatment of cutaneous symptoms [8]. While corticosteroids are often given following anaphylaxis, there is little evidence of immediate benefit or prevention of delayed biphasic reaction [9]. However, supportive therapy should never delay the administration of epinephrine and cannot replace epinephrine as the definitive treatment for anaphylaxis [8]. Delayed epinephrine remains the greatest risk factor for anaphylaxis mortality and should always be given with a low threshold to treat [8].

Trastuzumab has potentially tremendous benefits, and when hypersensitivity reactions occur, rechallenge with desensitization protocols has become more common and has been included in small numbers in several case series of desensitization to oncology treatments [10,11,12,13]. There are currently no formal guidelines or recommendations concerning trastuzumab rechallenge, but desensitization protocols have been published in the literature [11,12,13]. A recent European Academy of Allergy and Clinical Immunology (EAACI) position paper proposed an algorithm for the evaluation of hypersensitivity reactions to chemotherapeutic agents [14]. It recommends serum tryptase levels and immunologic skin testing for patients with immediate hypersensitivity reactions in order to assess their risk of continuing treatment [14]. If deemed low risk, patients can undergo a challenge in which the drug is administered in a monitored environment [14]. If hypersensitivity is suspected and the patient is deemed to have a moderate-to-high risk of reaction on further exposure, they can undergo rapid drug desensitization [14]. During desensitization, the drug is administered in diluted amounts according to a multi-step protocol that increases the target dose over time, resulting in a temporary state of tolerance [7]. While the mechanism is incompletely understood, this immunologic tolerization of the offending drug is induced by inhibiting mast cell activation responses and thus preventing anaphylaxis [15]. 

Oncology presents a unique situation in which repeat drug exposure after serious adverse reactions is often warranted due to the mortality risk of untreated cancer—allergists can assist with both symptom assessment and risk mitigation.

## 2. Case Presentation

We report successful desensitization in a 43-year-old female with locally advanced HER2-positive breast cancer following a severe anaphylactic reaction to trastuzumab infusion for which epinephrine was not administered. 

At initial assessment, the patient had biopsy-confirmed HER2-positive, ER/PR-negative, and right breast invasive mammary carcinoma measuring 1.8 cm in diameter. Imaging demonstrated multiple axillary lymph nodes (largest 3.0 × 2.1 cm) and a right supraclavicular node (1 cm). No metastases were identified. Her oncologist assessed this as stage III and recommended neoadjuvant Docetaxel, Carboplatin, and trastuzumab every 3 weeks for a total of 6 cycles, followed by mastectomy. Chemotherapy was administered at a local clinic in a northern region of the province of Manitoba. The patient was otherwise healthy, took no other medications, with no history of allergies or allergic disease.

Cycle 1 of neoadjuvant therapy was administered without issue. However, during Cycle 2, the patient experienced an adverse reaction approximately 2 min after trastuzumab infusion was initiated. She became flushed and reported evolving symptoms that quickly culminated in hypotension (BP 80/49), tachycardia (HR 147), dyspnea (O_2_ sat 91%), and nausea. She had not yet received Docetaxel or Cyclophosphamide. The trastuzumab infusion was discontinued, and her IV was flushed with normal saline (NS). O_2_ was applied at 6 L/min via a face mask. The patient received 50 mg Diphenhydramine IV. SpO_2_ continued to decrease, at which point O_2_ was increased to 10 L/min. Hydrocortisone, 100 mg IV, was then administered. The patient became unresponsive and began vomiting (total 500 mL emesis). Vital signs were persistently unstable, and multiple NS boluses were administered via pressure bag to a total of 5 L. She did not receive epinephrine. SpO_2_ stabilized on 10 L/min face mask and remained stable on a decrease to 5 L/min. The patient was observed for 2 additional hours. Each decrease in NS IV rate resulted in hypotension with BP of 80/52 and tachycardia of 120–150 bpm. The patient was unconscious for 1.5 h and was weaned off O_2_, at which point she was awake and walked to the bathroom with assistance from the nursing staff. On return to bed, her vital signs quickly deteriorated again. The local emergency department (ED) was contacted, and the patient was transferred for further management. 

After a presentation to the ED, the patient was given norepinephrine IV. This decision was made based on the large volume of IV fluid she had received prior to arrival. She was weaned off the norepinephrine and was stable and discharged home later that evening. A tryptase level was not drawn due to patient refusal. 

Manitoba has recently established a multidisciplinary Medical Oncology and Allergy Clinic aimed at allowing oncology patients to receive treatments despite adverse reactions. Re-administration with desensitization was pursued, as the anti-HER2 component to her therapy was felt to carry significant potential benefit. 

Cycle 2 was administered two weeks later with a desensitization protocol. Desensitization involves administering the drug at increasing concentrations and infusion rates with set time intervals to allow those with an anaphylactic allergy to receive the medication safely (Table 1) [16]. The patient tolerated the desensitization protocol for Cycle 2 and has since completed up to 5 cycles using desensitization without issue. 

## 3. Discussion

In this case, epinephrine was not administered. The patient developed a severe anaphylactic reaction and remained persistently hypotensive and hypoxic to the point of unresponsiveness despite the repeated use of supportive measures. The use of Diphenhydramine—a first-generation antihistamine—was likely detrimental, as it may have reduced consciousness and worsened hypotension; antihistamines do not save lives as they only treat mild pruritus and hives. Epinephrine was required to treat the bronchoconstriction and vasodilation secondary to anaphylactic shock, and the patient continued to deteriorate when she did not receive it.

This patient’s reaction to trastuzumab was anaphylaxis. While it may not have been absolutely clear at the time, the clinical uncertainty should not have prevented epinephrine from being administered. She was likely sensitized on first exposure when she tolerated the initial dose of trastuzumab and then reacted within minutes of starting her Cycle 2 infusion. Anaphylaxis is a clinical diagnosis, and she met the first diagnostic criteria for anaphylaxis [5] based on her acute development of generalized flush, hypotension, bronchospasm and hypoxia, and gastrointestinal symptoms. Furthermore, she failed to improve clinically despite receiving antihistamines, corticosteroids, and over 5 L of crystalloid intravenous fluids. This suggests vasodilation and distributive shock secondary to anaphylaxis mediators. After the reaction, the patient was referred to the Medical Oncology and Allergy Clinic for evaluation. The patient declined intradermal skin testing to confirm Trastuzumab hypersensitivity, as she understood that regardless of positive or negative results, she would proceed with desensitization, given the severity of her reaction. Immunologic skin-prick or intradermal testing is always recommended but is sometimes unavailable or does not change management. In the case of some chemotherapy agents, it may not be feasible due to cutaneous toxicity [15]. The tests are also poorly validated as there is limited information about their sensitivity and specificity [17]. There are currently no standardized skin-prick or intradermal testing doses for many chemotherapy agents and monoclonal antibody therapies [15]; some concentrations have been recommended, but the evidence is limited [17], including for trastuzumab [10]. Therefore, even when available, immunologic skin tests are not always reliable in confirming hypersensitivity after a reaction occurs. In this particular case of severe anaphylaxis, desensitization is the appropriate intervention for further oncologic therapy regardless of intradermal test results. The fact that she also successfully tolerated rechallenge with desensitization and was able to continue treatment reinforces anaphylaxis as the most likely etiology. 

Serum tryptase can also be retrospectively used to confirm anaphylaxis in the event of clinical uncertainty and is recommended by current guidelines [5]. While the patient did meet the clinical criteria for anaphylaxis [5], a serum tryptase was unfortunately not drawn due to the patient’s refusal, which is a disadvantage. The lack of baseline tryptase also means that confounding conditions, such as Mastocytosis and Hereditary Alpha Tryptasemia, could not be evaluated, especially given the severity of the patient’s reaction. However, genetic testing for Hereditary Alpha Tryptasemia is not currently available in Manitoba, locally, or as an external test. Furthermore, even if a Tryptase were drawn, a normal level would not have ruled out Mastocytosis, and the patient ultimately would have required a bone marrow biopsy to diagnose the condition [18]. The authors were reassured by the patient’s absent history of prior anaphylaxis or other symptoms indicative of mast cell disorder and felt that the condition was unlikely in this case. 

Delay in epinephrine administration is a common issue worldwide, particularly with drug-induced anaphylaxis. There is often failed or delayed recognition in hospital and emergency room settings, and even when recognized, epinephrine is under-used, and patients are inappropriately treated with only fluids and antihistamines [19,20]. One Canadian study postulated that healthcare provider concerns about epinephrine side effects were sometimes a barrier to its administration [19], but there are no absolute contraindications to the administration of epinephrine when treating anaphylaxis [8]. 

There are limited studies informing trastuzumab-specific guidelines around treatment protocols when adverse reactions occur. However, the current anaphylaxis guidelines for the diagnosis and treatment of suspected anaphylactic reactions apply to all instances of anaphylaxis [5]. This includes reactions to monoclonal antibody therapies, such as trastuzumab, chemotherapy agents, and other drugs [5]. The treatment guidelines should therefore be implemented in oncology settings, such as the one described in this case, and epinephrine should always be the recommended treatment. Multidisciplinary involvement in this and several similar cases has prompted a change in the CancerCare Manitoba treatment room protocols to ensure prompt administration of epinephrine intramuscularly at the onset of possible anaphylaxis.

When hypersensitivity reactions occur in response to chemotherapy agents or biologics, it can become a serious impediment to patients receiving first-line therapies. Drug desensitization is a means by which patients can tolerize agents that have previously caused hypersensitivity reactions [21]. The drug is typically administered using a series of gradually increasing dose increments that eventually total to equal the target drug dose [21]. Rechallenge with desensitization should be considered before therapy is discontinued or switched to potentially sub-optimal alternatives. 

The EAACI gave Task Force status to the interdisciplinary field of AllergoOncology in 2014, which aims to encourage the exchange of expertise in order to further understanding of both topics [14]. The field focuses on the relationship between allergic responses and cancer, immunomodulation, including IgE-mediated responses, and aims to provide new insights into treatments, such as cancer immunotherapies [14]. While allergic reaction to anti-cancer agents is not a new problem, the management of allergy and allergic disease in daily clinical oncology is an emerging area that falls under this interdisciplinary umbrella [14]. 

Reactions to chemotherapy agents and monoclonal antibody therapies are increasingly common in the last several decades and have become more clinically significant as the number and efficacy of therapies have increased [7]. While there is an abundant collaboration between allergists and oncologists in many European jurisdictions as a result of the EAACI’s efforts in the expanding field of AllergoOncology [14], there are only a handful of examples in North America. Only two such clinics have been established in the United States, out of the Brigham and Women’s Hospital in Boston and the Albert Einstein College of Medicine in New York. 

The Medical Oncology and Allergy Clinic in Winnipeg, Manitoba, is the first formal collaboration in Canada with one of its goals to provide recommendations regarding acute management of hypersensitivity reactions and prevention of their recurrence (e.g., with the administration via desensitization). When patients have adverse reactions to their cancer therapies, they are promptly referred to the clinic for urgent assessment, immunologic skin testing, and consideration of rechallenge or desensitization. The clinic deals with all allergy-related issues experienced by current oncology patients. This includes not only infusion-related or anaphylactic reactions and subsequent skin testing and desensitization but also suspected allergic reactions to oral anti-cancer drugs and contrast dye. Patients with penicillin or other antibiotic allergies are also seen, especially those being considered for allogenic stem cell transplant. Prior to the clinic’s establishment, local allergists and oncologists noticed there was a high frequency of often time-sensitive consultations for oncology patients. This led to the involvement of the hospital’s Chief Medical Officer, department heads of medical oncology and allergy, and directors of the Systemic Therapies program and Provincial Oncology Drug Program. The data supporting allergist involvement in medical oncology was reviewed, along with the regular consults, leading to the agreement that a dedicated clinic would then provide the time, place, and staff to prioritize the management of oncology patients’ allergy-related problems. The clinic was established in September 2022 and runs one half-day per week, and approximately five patients are seen per clinic. The timing of referral is generally quick, and patients are seen within 5 business days. 

In the case of this patient, she was able to continue Cycle 2 of trastuzumab after a mere 2-week delay. She went on to complete the full course without further adverse reactions and an apparent positive clinical response to her treatment regimen. She may have had worse outcomes if she had not undergone desensitization and if trastuzumab had been discontinued. 

## 4. Conclusions

Treatment for anaphylaxis is epinephrine: the largest risk factor for anaphylaxis-related death is delayed epinephrine. Antihistamines are not life-saving, and steroids have limited utility in anaphylaxis treatment. Authors are currently changing Manitoba-wide treatment protocols to ensure early recognition and appropriate treatment of anaphylaxis during treatment administration. The authors eagerly encourage oncology programs across the country to reassess their protocols for the treatment of adverse reactions.

Hypersensitivity reaction to anti-cancer drugs should prompt urgent allergy referral for assessment and consideration of risk-mitigating procedures (e.g., desensitization) prior to discarding life-saving treatments. We propose this multidisciplinary clinic model as a treatment framework moving forward, with the goal of continuing first-line therapies in cancer patients who develop drug-hypersensitivity. This case highlights the unmet need for a multidisciplinary approach to the management of oncology patients who experience hypersensitivity reactions.

## Figures and Tables

**Table 1:** Trastuzumab Desensitization Protocol [16].

1% of the total dose (BAG 1)	IV in NS 250 mL following the administration rates below. Step 1: 2 mL/h for 15 min, then Step 2: 5 mL/h for 15 min, then Step 3: 10 mL/h for 15 min, then Step 4: 20 mL/h for 15 min. Once Step 4 is complete, discard the remainder of the bag and proceed immediately to BAG 2.
10% of the total dose (BAG 2)	IV in NS 250 mL following the administration rates below. Step 5: 5 mL/h for 15 min, then Step 6: 10 mL/h for 15 min, then Step 7: 20 mL/h for 15 min, then Step 8: 40 mL/h for 15 min. Once Step 8 is complete, discard the remainder of the bag and proceed immediately to BAG 2.
99.2% of the total dose (BAG 3)	IV in NS 250 mL following the administration rates below. Step 9: 10 mL/h for 15 min, then Step 10: 20 mL/h for 15 min, then Step 11: 40 mL/h for 15 min, then Step 12: 80 mL/hour until the infusion is complete.

## Data Availability

The data presented in this study are available in this article.
